# Comparing alternative approaches to measuring the geographical accessibility of urban health services: Distance types and aggregation-error issues

**DOI:** 10.1186/1476-072X-7-7

**Published:** 2008-02-18

**Authors:** Philippe Apparicio, Mohamed Abdelmajid, Mylène Riva, Richard Shearmur

**Affiliations:** 1 Spatial Analysis and Regional Economics Laboratory, Université du Québec, Institut national de la recherche scientifique, Urbanisation, Culture et Société, 385 rue Sherbrooke est, Montréal (Québec), H2X 1E3, Canada; 2 Department of Geography, Université du Québec à Montréal, Pavillon Hubert-Aquin, 1255 rue Saint-Denis, Montréal (Québec), H2X 3R9, Canada; 3 Department of Social and Preventive Medicine, Faculty of Medicine, University of Montréal, P.O. Box 6128, Downtown Station, Montréal (Québec), H3C 3J7, Canada

## Abstract

**Background:**

Over the past two decades, geographical accessibility of urban resources for population living in residential areas has received an increased focus in urban health studies. Operationalising and computing geographical accessibility measures depend on a set of four parameters, namely definition of residential areas, a method of aggregation, a measure of accessibility, and a type of distance. Yet, the choice of these parameters may potentially generate different results leading to significant measurement errors.

The aim of this paper is to compare discrepancies in results for geographical accessibility of selected health care services for residential areas (i.e. census tracts) computed using different distance types and aggregation methods.

**Results:**

First, the comparison of distance types demonstrates that Cartesian distances (Euclidean and Manhattan distances) are strongly correlated with more accurate network distances (shortest network and shortest network time distances) across the metropolitan area (Pearson correlation greater than 0.95). However, important local variations in correlation between Cartesian and network distances were observed notably in suburban areas where Cartesian distances were less precise.

Second, the choice of the aggregation method is also important: in comparison to the most accurate aggregation method (population-weighted mean of the accessibility measure for census blocks within census tracts), accessibility measures computed from census tract centroids, though not inaccurate, yield important measurement errors for 5% to 10% of census tracts.

**Conclusion:**

Although errors associated to the choice of distance types and aggregation method are only important for about 10% of census tracts located mainly in suburban areas, we should not avoid using the best estimation method possible for evaluating geographical accessibility. This is especially so if these measures are to be included as a dimension of the built environment in studies investigating residential area effects on health. If these measures are not sufficiently precise, this could lead to errors or lack of precision in the estimation of residential area effects on health.

## Background

In recent urban health studies, an increased focus has been directed to evaluating the accessibility of urban resources, either for individuals or for populations living in residential areas. Although the concept of "accessibility" is multidimensional (accessibility may be defined in terms of affordability, acceptability, availability and spatial accessibility [1]) evaluating geographical accessibility in residential areas offers critical information for public policy in planning and service provision as it allows for the identification of areas with lower (or higher) access to urban resources and the assessment of spatial and social inequalities in access [2,3].

Geographical accessibility refers to the ease with which residents of a given area can reach services and facilities [2]. Most common approaches for defining geographical accessibility are based on distance or travel time to a resource (for a review, please refer to [4]). These measures assume that every member of the population is a potential user of the service; the pattern of spatial accessibility will depend on the relative location of the population and services [5,6]. Table 1 synthesises approaches for conceptualizing and measuring different dimensions of geographical accessibility.

**Table 1:** Approaches for conceptualizing and measuring the geographical accessibility of services and facilities for residential areas

**Conceptualization**	**Accessibility measures**
Immediate proximity	The distance between a location and the closest facility
Availability within one area unit	The number of facilities contained within a given unit (for example, census tract)
Availability provided by the immediate surroundings	The number of facilities within a given distance from a point of origin
Average cost to reach all destinations	The average distance between a location and all facilities
Average cost to reach diversity	The average distance between a location and *n *facilities

Adapted from Talen [41] and Apparicio et al. [32].

Several studies have measured the geographical accessibility in residential areas of services and facilities that have the potential to contribute to the population's well-being and health such as health care services [2,7-18], recreational facilities [2,16,18,19], and food supermarkets [16,18,20-23]. Accessibility to these types of resources is especially important for populations with limited mobility and revenue since more direct and easier access confers opportunities by reducing the time and financial costs of access, and by potentially influencing life choices [24]. Other studies have measured geographical accessibility of resources potentially associated with more negative health outcomes, such as waste facilities, fast food restaurants, and pollution from large motorways [25-27].

Over the past two decades, the operationalization of geographical accessibility measures in urban and health studies has become easier, largely due to developments in GIS transportation softwares and modules. These measures require the specification of a set of four parameters, namely 1) a spatial unit of reference for the population, i.e. a definition of residential areas; 2) an aggregation method, i.e. to account for the distribution of population in the residential area; 3) a measure of accessibility; and 4) a type of distance for computing the accessibility measures selected. The choice of these parameters is likely to generate different results, potentially leading to significant measurement errors [2,28,29].

In this paper, we investigate differences in results when geographical accessibility of residential areas (census tracts) to health care services is computed using different distances types and different aggregation methods. In the next section, we describe methods for defining the four parameters for computing accessibility measures. With these methods in mind, we then provide an overview of methodological issues in measuring accessibility.

### Evaluating geographical accessibility of services and facilities in residential areas: specifying a set of parameters

#### Spatial unit of reference and aggregation methods

Selecting the appropriate spatial unit of analysis, i.e. the operational definition for residential areas, is critical for minimizing aggregation errors [2,30]. Aggregation error arises from the distribution of individuals around the centroid of spatial units [2]. As spatial units vary in size from smaller areas, e.g. census blocks, to larger ones, e.g. census tracts, accessibility measured for smaller units is less subject to aggregation error than that measured for larger spatial units [2].

**Figure 1:**
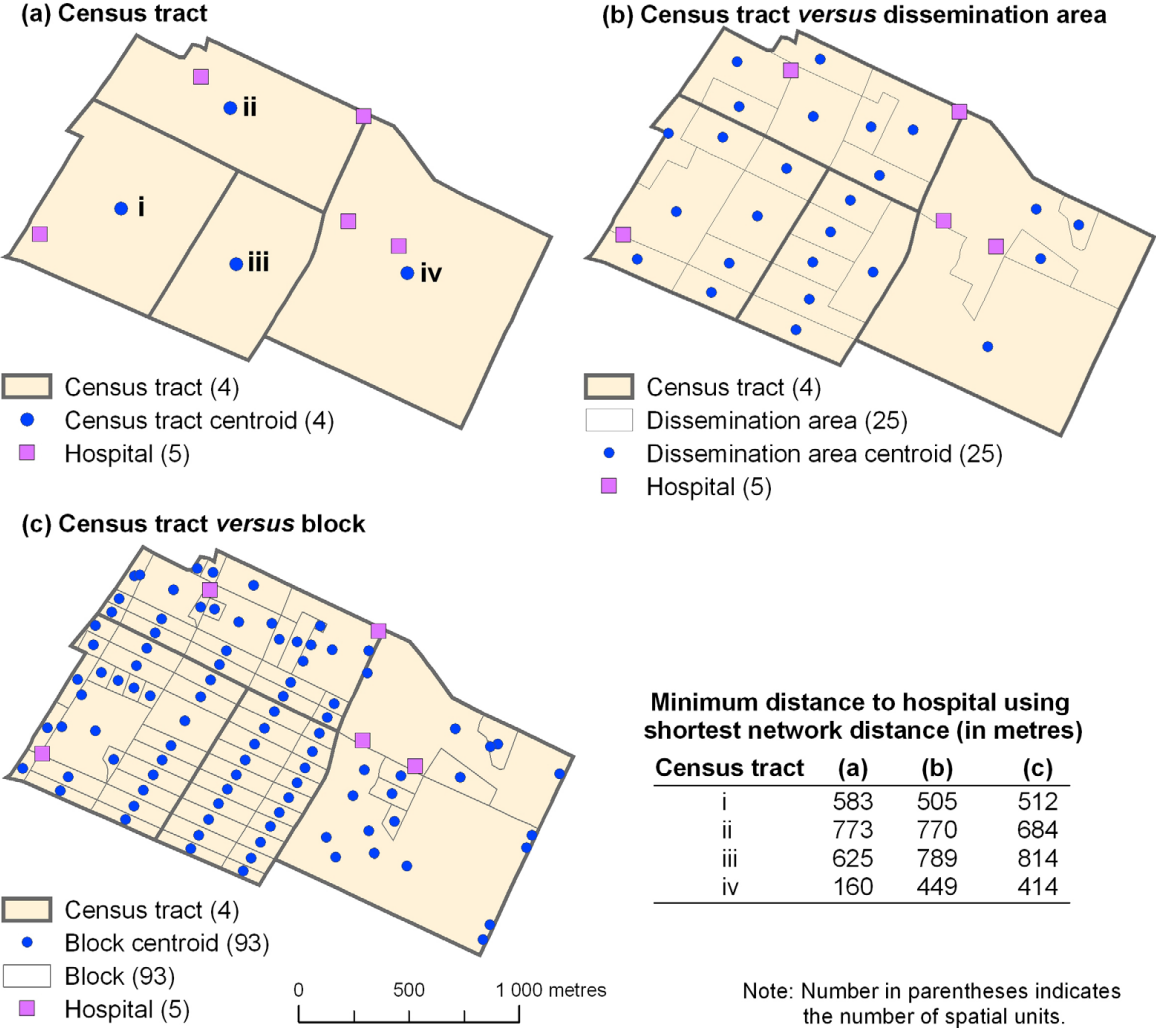
Choosing the spatial unit of reference for calculating distances and error aggregation.

To evaluate the geographical accessibility of a service for a population living in a residential area, e.g. a census tract, three methods can be used [2]; they are illustrated in Figure 1. The first method consists in computing the distance between the centroid of the census tract and the service (Figure 1.a). This method shows the inappropriateness of ignoring the spatial distribution of the population inside the census tract [2].

The second method consists of calculating the population-weighted mean centre of the census tracts (Equation 1) and then evaluating the distance between this new location and the service. Toward this end, smaller spatial units entirely contained by the census tracts can be used, such as dissemination areas, census blocks, or postal codes. This method accounts for the spatial distribution of the population inside the census tract in order to minimize aggregation error.

$$\left(\bar{x_{i}},\bar{y_{i}}\right)=\left(\frac{\sum_{b\in i}w_{b}x_{b}}{\sum_{b\in i}w_{b}},\frac{\sum_{k\in i}w_{b}y_{b}}{\sum_{b\in i}w_{b}}\right)$$

Where:

*w*_*b *_= total population of spatial unit *b *completely within census tract *i *(i.e. dissemination area or census block or postal code).

*x*_*b *_and *y*_*b *_= *X *and *Y *coordinates of spatial unit *b*.

Finally, the third method consists of computing the distance between the services and each centroid of spatial units completely within census tracts, and then calculating the average of these distances weighted by the total population of each unit (Figure 1.b and 1c). In comparison with the previous methods, this one is more accurate because it more exactly accounts for the distribution of the population inside the census tract.

#### Accessibility measures

The five most commonly used measures of accessibility are: 1) the distance to the closest service, 2) the number of services within *n *metres or minutes, 3) the mean distance to all services, 4) the mean distance to *n *closest services, and 5) the gravity model. If the more accurate aggregation method detailed previously is selected, these accessibility measures can be written as:

$$Z_{i}^{a}=\frac{\sum_{b\in i}w_{b}(\min\left|d_{bs}\right|)}{\sum_{b\in i}w_{b}},$$

Where:

$Z_{i}^{a}$ = mean distance between census tract *i *and closest service.

*w*_*b *_= total population of spatial unit *b *completely within census tract *i*.

*d*_*bs *_= distance between spatial unit *b *and service *s*.

$$Z_{i}^{b}=\frac{\sum_{b\in i}W_{b}\sum_{j\in S}S_{j}}{\sum_{b\in i}W_{b}},$$

Where:

$Z_{i}^{b}$ = mean number of services within *n *metres or minutes of census tract *i*.

*w*_*b *_= total population of spatial unit *b *completely within census tract *i*.

*S *= all services.

*S*_*j *_= number of services within *n *metres or minutes of spatial unit centroid *b *with *S*_*j *_= 1 where *d*_*bs *_≤ *n *and *S*_*j *_= 0 where *d*_*bs *_> *n*.

$$Z_{i}^{c}=\frac{\sum_{b\in i}w_{b}d_{bs}}{\sum_{b\in i}w_{b}},$$

$Z_{i}^{c}$ = mean distance between census tract *i *population and all services.

*w*_*b *_= total population of spatial unit *b *completely within census tract *i*.

*d*_*bs *_= distance between spatial unit centroid *b *and service *s*.

$$Z_{i}^{d}=\frac{\sum_{b\in i}w_{b}\sum_{s}\frac{d_{bs}}{n}}{\sum_{b\in i}w_{b}},$$

$Z_{i}^{d}$ = mean distance between census tract *i *and *n *closest services.

*w*_*b *_= total population of spatial unit *b *completely within census tract *i*.

*d*_*bs *_= distance between spatial unit centroid *b *and service *s, d*_*bs *_is sorted in ascending order.

*n *= number of closest services to be included in measure.

$$Z_{i}^{e}=\frac{\sum_{b\in i}w_{b}\sum_{S}S_{ws}d_{bs}^{-\alpha }}{\sum_{b\in i}w_{b}},$$

$Z_{i}^{e}$ = mean value of potential gravity.

*w*_*b *_= total population of spatial unit *b *completely within census tract *i*.

*S *= number of services in study area.

*d*_*bs *_= distance between spatial unit centroid *b *and service *s*.

*α *= friction parameter (usually 1, 1.5 or 2).

*S*_*ws *_= weight given to the service *s *such as its size (for example, number of beds for a hospital).

#### Types of distance

**Figure 2:**
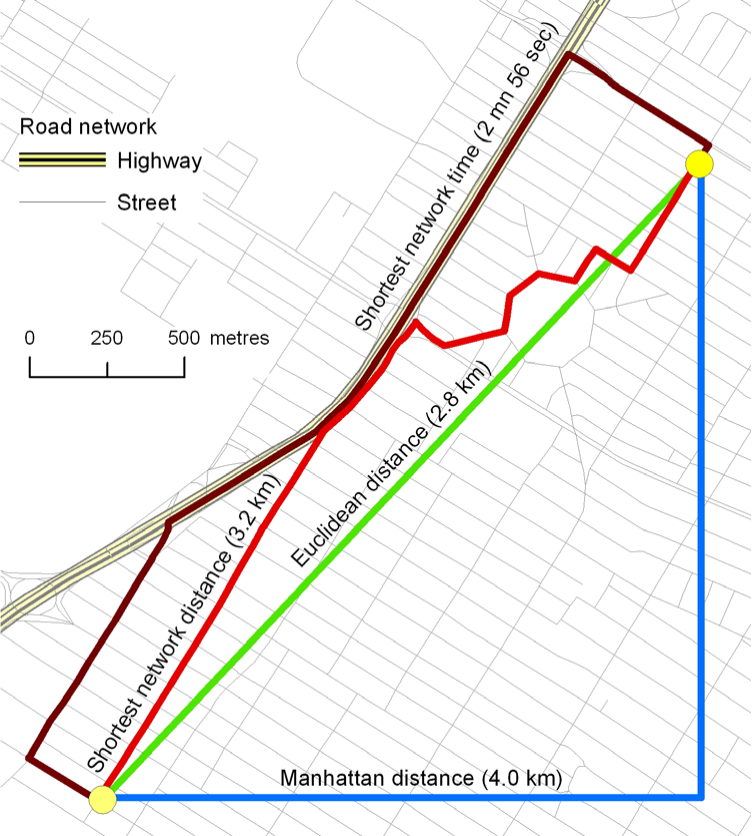
Several types of distance.

Four types of distance are typically used for calculating accessibility measures: Euclidean distance (straight-line), Manhattan distance (distance along two sides of a right-angled triangle opposed to the hypotenuse), shortest network distance and shortest network time (Figure 2) [28,31]. Euclidean and Manhattan distances can easily be computed using geographic coordinates:

$$d_{ij}=\sqrt{{\left(x_{i}-x_{j}\right)}^{2}+{\left(y_{i}-y_{j}\right)}^{2}},$$

*d*_*ij *_= |*x*_*i *_- *x*_*j*_| + |*y*_*i *_- *y*_*j*_|,

Where:

*X*_*i *_*and Y*_*i *_= X and Y coordinates of point *i *with a plane projection.

On the other hand, calculation of network distance and network time distance – which represent respectively the shortest and fastest paths between two points using a street network – is more complex. Indeed, the computation of these two distances necessitates geometric network files – with directions, speed limits, turning restrictions, and delays available for each street segment – integrated into GIS, and a GIS module or GIS software specialized in transportation analysis (ESRI Network Analyst Extension or TransCad software, for example).

Shortest network and network time distances represent two different objectives. Shortest network distance is useful for evaluating the path between two points as if taken on foot; consequently, it is frequently used in studies on the accessibility of "proximal" services and facilities [4,18,20,32-34]. Shortest time distance is more accurate for evaluating distances by car or public transportation.

### Methodological issues and accuracy in measuring geographical accessibility

When evaluating geographical accessibility, the choice of these parameters is likely to generate different results, potentially leading to significant measurement errors [2,28,29].

Most studies examining the geographical accessibility of health care and health-related services have been concerned with measuring the accessibility of the closest facility using Euclidean distance [2,13], shortest network distance [8,18,32], shortest network time distance [9,11,16,17,35], or a combination of distance types [10,12,15]. Others have also examined proximity to diversity by measuring the average shortest network distance to selected services [32], and the offer provided by the immediate surroundings, i.e. of the number of services within a given distance [7,14,18,32]. Few studies have conceptualized different dimensions of geographical accessibility within one investigation (for exceptions, see [14,15,32]), although this would be useful in order to describe the complexity of geographical accessibility of a given service [32]. Furthermore, within a given set of data, the choice of the accessibility measure is fundamental since accessibility varies depending on the indicator used [3,4,36].

Some studies have compared discrepancies in results when geographical accessibility was measured using different types of distances. In a study exploring trade-offs between various types of distance, Apparicio and colleagues [28] compared distance matrices based on simple (Euclidean, Manhattan) and network (road, time) measures of distance between all census tracts in Canada's eight largest metropolitan areas. They examined whether the Euclidean and Manhattan approximations are correlated with a more accurate measure of distance, i.e. travel time along the road network, at the metropolitan and census tract levels. The authors concluded that, at the metropolitan level, the use of Euclidean or Manhattan distances to estimate shortest network time does not introduce major errors. However, in sub-metropolitan areas, or areas located away from the central business district, the use of Euclidean or Manhattan approximations of shortest network time may lead to substantial errors. In measuring accessibility in these areas, network-based distance/time matrices may provide more appropriate results. Similar results were also observed [10,12,15].

With respect to operational definition of residential areas, a wide variety of area units has been used, ranging from smaller units such as census meshblocks [9,10,16,18], enumeration districts [11,13] and census output areas [12] to larger units such as census tracts [7,17,32,35], communities and city-defined neighbourhoods [2,8], and wards [13-15]. Some studies have controlled for the location of the population within the spatial unit by calculating the population-weighted mean centre of the spatial unit [12,14,16,17,35] or by calculating the population-weighted mean accessibility of smaller spatial units located within the spatial unit of interest [2,13,32]. Nonetheless, a considerable number of studies do not employ methods for minimizing aggregation errors, i.e. they compute accessibility for the geometric centroid of the spatial unit.

For public policy and planning, measuring geographical accessibility of urban resources and facilities is of interest as it conveys information on the presence of enabling resources [37] or opportunity structures [38] in the residential environment. Geographical accessibility measures are however prone to a variety of methodological problems, among which is error induced by using different distance types [28] and aggregation methods [2,29].

### Study objectives

In this paper, we investigate differences in results when geographical accessibility of residential areas (census tracts) to selected health care services is computed using different distances types and different aggregation methods. Specific objectives are to: 1) Compare measures of distances; and 2) Estimate aggregation errors for several accessibility measures.

## Data and methods

### Study area and health services

**Figure 3:**
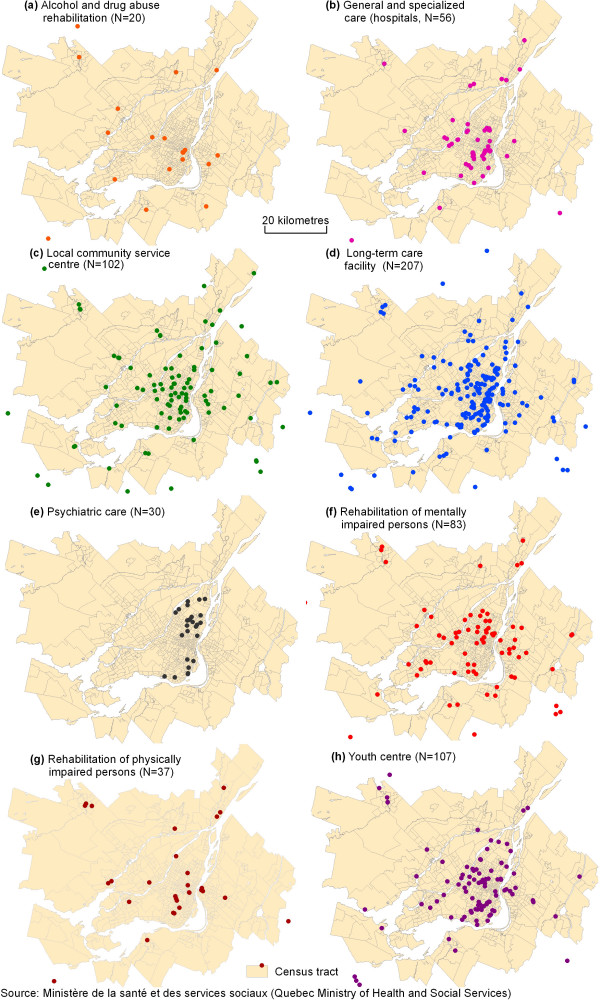
Categories of health services for the Montréal CMA, 2006.

This study focuses on the Montréal census metropolitan area (CMA) which has a population of about 3.4 million inhabitants. The territory of the Montréal CMA is divided into 852 census tracts, 5,829 dissemination areas and 25,767 blocks with respective average population sizes of 4,022, 588 and 133 inhabitants, as defined by Statistics Canada. A total of 642 health services grouped into eight categories were integrated into geographic information systems (ArcGis) by geocoding addresses (Figure 3). These health services were inventoried from the website of the Ministère de la santé et des services sociaux du Québec (Quebec Ministry of Health and Social Services). Of the 642 services, 65 are located in a 10 km buffer zone around the boundaries of the Montréal CMA. These 65 services were included in order to prevent underestimation of accessibility in the bordering zones of the Montréal CMA.

### Comparing distance types

To explore variations in results according to distance type, we calculate the four distance types – Euclidean, Manhattan, shortest network path and shortest network time distances – between the 642 health services and the centroids of census tracts, dissemination areas and blocks. In total, more than 83 million distances are computed (Table 2), with SAS software for Euclidean and Manhattan distances, and with the Network Analyst Extension of ArcView 3.3 [39] by using CanMap Streetfiles from DMTI [40] for shortest network and shortest network time distances.

**Table 2:** Distances calculated between health services and spatial units

Spatial units (origins)		Health services (destinations)	Types of distance*	Distances calculated
Type	N			
Census tracts	852	642	4	2,187,936
Dissemination areas	5,829	642	4	14,968,872
Blocks	25,767	642	4	66,169,656

Total	32,448	642	4	83,326,464

* Euclidean, Manhattan, shortest network distance, shortest network time.

Once these four distance types are computed, correlation analyses are performed globally and locally across entire census tract, dissemination area and block matrices. First, the global analysis, which yields one value for the CMA as a whole, allows us to assess the degree of correlation between the four distance types. Then, we examine correlations between the four distances for each spatial unit centroids and the 642 health services. This local analysis stage yields one mappable value for each census tract, dissemination area and block and allows to identify spatial variation in the degree of correlation between the four distance types.

### Evaluating aggregation errors when measuring geographical accessibility

The same approach, i.e. global and local analyses, was used to evaluate aggregation errors for several accessibility measures at the census tract level. The global analysis involves calculating correlations between three aggregation methods: 1) the census tract centroid; 2) the population-weighted mean of the accessibility measure for dissemination areas within census tracts; and 3) the population-weighted mean of the accessibility measure for blocks within census tracts, the most accurate method. Although accessibility was computed for each of the eight categories of health services, for purposes of conciseness, results are reported only for accessibility of general and specialized care i.e. hospitals (n = 56) for census tracts. It is worth noting, that similar patterns of correlation were observed for other health services.

## Results

### Correlations between the four types of distances

#### Global correlations

Table 3 presents results for global correlation coefficients between the four types of distances computed for the entire sample of health services (n = 642). Three observations can be made. First, at the metropolitan scale, independently of the type of distance used, results are globally similar as indicated by high correlation coefficient values (greater than 0.95). Second, in comparison with Manhattan distance, Euclidean distance is most strongly correlated with the more accurate network path and time distances. Thus, if it is impossible to compute network distance in a study focussing on geographical accessibility in the Montréal CMA, Euclidean distance seems preferable to Manhattan distance. These results are in line with those of Apparicio et al. [28] for eight Canadian metropolitan areas (Toronto, Montréal, Vancouver, Ottawa-Hull, Calgary, Edmonton, Québec and Winnipeg), and with those of Fone et al. [12] for Caerphilly county borough in Wales. Finally, as expected, correlations between both network distances are almost perfect (0.992). This means that if directions and speed limits are unknown for computing the shortest network time, the shortest network distance is a very reliable alternative.

**Table 3:** Global Pearson correlations between alternative types of distance

	Cartesian system		Shortest network	
Distance	Euclidean	Manhattan	Distance	Time
Distances between census tracts and health services (N)	546,984	546,984	546,984	546,984
Euclidean	1.000			
Manhattan	0.987	1.000		
Distance	0.988	0.971	1.000	
Time	0.976	0.959	0.992	1.000
Distances between dissemination areas and health services (N)	3,742,218	3,742,218	3,742,218	3,742,218
Euclidean	1.000			
Manhattan	0.986	1.000		
Distance	0.987	0.969	1.000	
Time	0.975	0.957	0.992	1.000
Distances between blocks and health services (N)	16,542,414	16,542,414	16,542,414	16,542,414
Euclidean	1.000			
Manhattan	0.985	1.000		
Distance	0.984	0.964	1.000	
Time	0.970	0.950	0.992	1.000

Note: All coefficient values are significant at the p < 0.0001 level.

#### Local correlations

**Figure 4:**
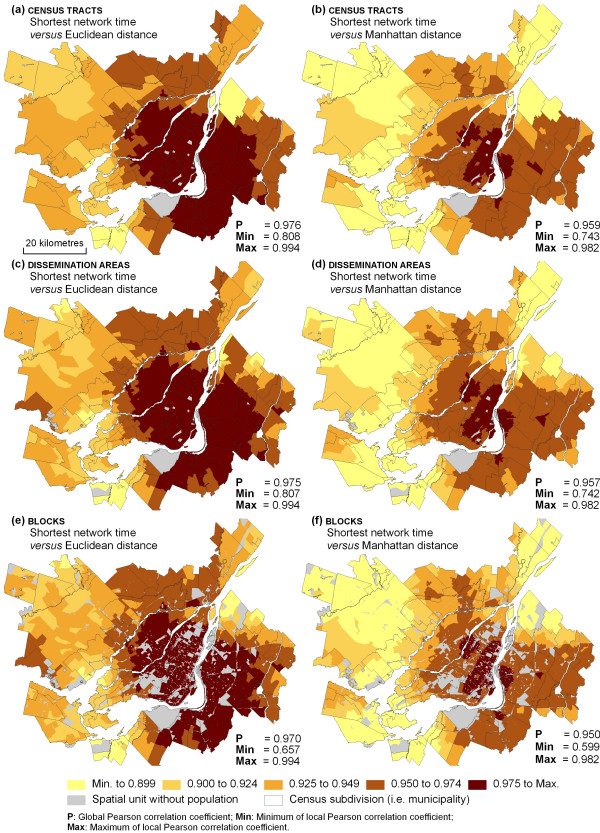
Comparing alternative types of distance between spatial units and health services using local Pearson correlations.

Although global correlations are high, they are not perfect (values differ from one). For this reason, local variations at the intra-metropolitan scale must exist and should be examined in detail. Figure 4 presents local Pearson coefficients between Euclidean distance and shortest network time, and between Euclidean and Manhattan distances for the geographical accessibility of the 642 health services computed from the centroids of census tracts, dissemination areas, and blocks.

Results show similar spatial patterns for the three spatial scales (census tract, dissemination area and block): with increasing distance from the central business district, correlations are reduced between Euclidean distance and shortest network time, and between Euclidean and Manhattan distances. For all spatial units in the centre of the Island of Montréal and those located on the south shore, correlations are higher. For those located on the periphery of the CMA, notably on the north shore, characterized by suburban areas, correlations are weaker, often lower than 0.9.

These results illustrate that for the Island of Montréal, integrating Euclidean distances at the census tract, dissemination area and block levels into statistical analysis, e.g. in regression or multilevel analysis, would yield similar results to those obtained if network distances were computed. However, if the focus is on the CMA as a whole, or on specific parts of the CMA, namely, those located in the northern suburbs, then results are likely to vary as a function of the distance type used to compute geographical accessibility.

### Aggregation errors

#### Global errors

The global analysis of aggregation errors is performed by means of Spearman correlations between the three methods of aggregation used to calculated 20 accessibility measures at the census tract level using the more accurate distances (network distances). Results are shown in Table 4 for hospitals only, although similar patterns of correlation were observed for other health services.

**Table 4:** Spearman rank correlations between measures of the accessibility of hospitals by aggregation method

		Accessibility measures using shortest network distance			Accessibility measures using shortest network time		
Hospitals		CTC^a^	WDA^b^	WBl^c^	CTC^a^	WDA^b^	WBl^c^
Minimum network distance	CTC^a^	1.000			1.000		
	WDA^b^	0.991	1.000		0.987	1.000	0.997
	WBl^c^	0.989	0.997	1.000	0.984	0.997	1.000
Average distance to three closest services	CTC^a^	1.000			1.000		
	WDA^b^	0.997	1.000		0.995	1.000	
	WBl^c^	0.997	0.999	1.000	0.994	0.999	1.000
Average distance to five closest services	CTC^a^	1.000			1.000		
	WDA^b^	0.998	1.000		0.996	1.000	
	WBl^c^	0.998	0.999	1.000	0.995	0.999	1.000
Average distance to all services	CTC^a^	1.000			1.000		
	WDA^b^	0.998	1.000		0.995	1.000	
	WBl^c^	0.999	0.999	1.000	0.995	0.998	1.000
Number of services within 500 metres	CTC^a^	1.000			1.000		
	WDA^b^	0.604	1.000		0.993	1.000	
	WBl^c^	0.588	0.838	1.000	0.992	0.999	1.000
Number of services within 1000 metres	CTC^a^	1.000			1.000		
	WDA^b^	0.828	1.000		0.990	1.000	
	WBl^c^	0.776	0.924	1.000	0.990	0.998	1.000
Number of services within 2000 metres	CTC^a^	1.000			1.000		
	WDA^b^	0.916	1.000		0.978	1.000	
	WBl^c^	0.898	0.968	1.000	0.976	0.996	1.000
Gravity model with α = 1	CTC^a^	1.000			1.000		
	WDA^b^	0.994	1.000		0.992	1.000	
	WBl^c^	0.993	0.998	1.000	0.990	0.997	1.000
Gravity model with α = 1.5	CTC^a^	1.000			1.000		
	WDA^b^	0.993	1.000		0.990	1.000	
	WBl^c^	0.992	0.996	1.000	0.988	0.996	1.000
Gravity model with α = 2	CTC^a^	1.000			1.000		
	WDA^b^	0.991	1.000		0.987	1.000	
	WBl^c^	0.991	0.995	1.000	0.986	0.994	1.000

^a ^Aggregation method based on census tract centroid (the least accurate method).

^b ^Aggregation method based on the population-weighted mean of the accessibility measure for dissemination areas within census tracts.

^c ^Aggregation method based on the population-weighted mean of the accessibility measure for blocks within census tracts (the most accurate method).

Correlations between the three aggregation methods are high (>0.9) for all measures of accessibility except for the number of services within 500, 1000 and 2000 metres. For example, correlation between the least exact aggregation method (census tract centroid) and the most exact based on blocks within census tracts is 0.588 for the number of hospitals within 500 metres, 0.776 for those within 1000 metres, and 0.898 for those within 2000 metres. This means that if we want to assess service provision in a close-proximity area around a census tract, it is preferable to use an aggregation method that precisely accounts for the distribution of population within it; if not, the risk of error might be considerable.

#### Local errors

A second stage of comparison of aggregation methods consists in assessing the absolute difference between the geographical accessibility results obtained from the methods based on census tract and dissemination areas centroids in relation to the most accurate method based on blocks within census tracts. The descriptive statistics for local errors are reported in Table 5 for hospitals.

**Table 5:** Aggregation errors in measures of the accessibility of hospitals at the census tract level

**Absolute difference between accessibility measure obtained from CTC^a ^and WBl^c ^aggregation methods**		Percentiles						
	Mean	5%	10%	25%	50%	75%	90%	95%
Shortest network distance								
Minimum network distance	365.35	11.19	22.54	57.40	147.23	365.04	947.76	1,595.45
Average distance to 3 closest services	284.19	5.88	12.07	32.04	102.51	276.01	719.27	1,250.98
Average distance to 5 closest services	290.26	5.10	11.81	34.16	94.54	275.32	800.69	1,300.49
Average distance to all services	309.02	3.44	7.42	26.94	89.12	275.50	869.61	1,470.33
Number of services within 500 metres	0.04	0.00	0.00	0.00	0.00	0.00	0.06	0.34
Number of services within 1000 metres	0.07	0.00	0.00	0.00	0.00	0.00	0.26	0.50
Number of services within 2000 metres	0.14	0.00	0.00	0.00	0.00	0.15	0.47	0.77
Shortest time distance								
Minimum time distance	0.37	0.02	0.03	0.09	0.20	0.41	0.84	1.34
Average distance to 3 closest services	0.31	0.01	0.02	0.06	0.15	0.34	0.73	1.11
Average distance to 5 closest services	0.31	0.01	0.02	0.06	0.15	0.35	0.75	1.16
Average distance to all services	0.32	0.02	0.03	0.06	0.15	0.37	0.73	1.22
Number of services within 10 minutes	0.69	0.00	0.00	0.07	0.39	0.93	1.72	2.60
Number of services within 20 minutes	0.76	0.00	0.00	0.00	0.25	0.71	1.98	3.20
Number of services within 30 minutes	0.52	0.00	0.00	0.00	0.04	0.41	0.98	2.15

**Absolute difference between accessibility measure obtained from WDA^b ^and WBl^c ^aggregation methods**	Mean	5%	10%	25%	50%	75%	90%	95%

Shortest network distance								
Minimum network distance	134.15	3.51	6.89	20.20	51.74	136.36	311.82	501.59
Average distance to 3 closest services	118.06	2.14	4.50	16.10	46.43	126.23	275.19	413.62
Average distance to 5 closest services	123.87	2.15	5.01	14.99	48.60	140.36	300.31	421.81
Average distance to all services	157.70	1.95	4.05	17.45	66.96	240.45	360.42	473.07
Number of services within 500 metres	0.02	0.00	0.00	0.00	0.00	0.00	0.03	0.12
Number of services within 1000 metres	0.04	0.00	0.00	0.00	0.00	0.00	0.08	0.22
Number of services within 2000 metres	0.08	0.00	0.00	0.00	0.00	0.05	0.22	0.40
Shortest time distance								
Minimum time distance	0.12	0.00	0.01	0.02	0.06	0.11	0.27	0.43
Average distance to 3 closest services	0.10	0.00	0.01	0.02	0.05	0.10	0.23	0.39
Average distance to 5 closest services	0.10	0.00	0.01	0.02	0.04	0.10	0.22	0.39
Average distance to all services	0.10	0.00	0.01	0.02	0.04	0.10	0.22	0.39
Number of services within 10 minutes	0.23	0.00	0.00	0.02	0.12	0.29	0.57	0.84
Number of services within 20 minutes	0.22	0.00	0.00	0.00	0.06	0.20	0.44	0.85
Number of services within 30 minutes	0.14	0.00	0.00	0.00	0.01	0.11	0.31	0.68

^a ^Aggregation method based on census tract centroid (the least accurate method).

^b ^Aggregation method based on the population-weighted mean of the accessibility measure for dissemination areas within census tracts.

^c ^Aggregation method based on the population-weighted mean of the accessibility measure for blocks within census tracts (the most accurate method).

Not surprisingly, the local errors are on the whole quite small, though not insignificant: for example, compared with the most accurate method, the census tract centroid method misestimates the distance to the closest hospital by an average of 365 m, and the dissemination area method by an average of 134 m. Up to the third quartile (75%), the local errors are still quite small: for 75% of census tracts, the error associated with the census tract centroid approach is less than 365 m. However, in 10% of cases, the error is greater than 948 m, and in 5% of census tracts the error is greater than 1.5 km (Table 5). Despite the high correlations, significant errors in the measurement of geographical accessibility can occur in a small number of cases.

**Figure 5:**
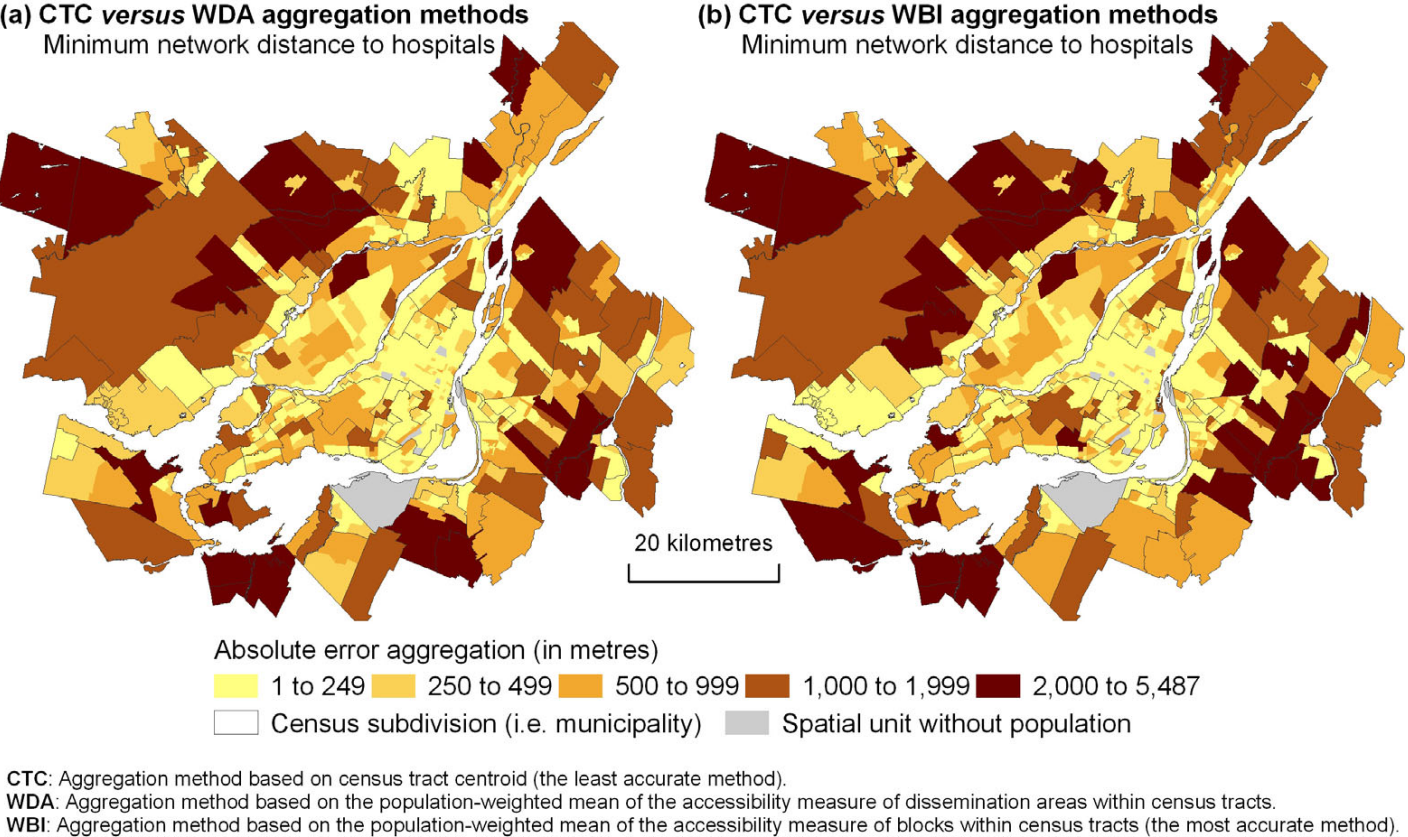
Evaluating local aggregation errors for hospitals.

Absolute differences between aggregation methods for the closest hospital computed using shortest network distance and shortest distance time are further mapped in Figure 5. Again, stronger absolute aggregation errors are observed in suburban areas on the south and north shores of the CMA; errors remain smaller in central areas of the Island of Montréal.

For the purposes of statistical studies at a general level, the least precise aggregation method – based on census tract centroids – is adequate: it enables the broad identification of areas in Montréal that have the least access to health services. However, if one wishes to reach more precise conclusions for specific neighbourhoods, major errors appear for 5% to 10% of census tracts.

## Conclusion

Over the past two decades, an increasing number of health studies have integrated the geographical accessibility of services and facilities as an important dimension of the built urban environment. The development of GIS with transportation module (ESRI Network Analyst Extension, for example) has largely fostered this increase. However, the results reported in this paper show that measures of geographical accessibility of urban health services may vary according to the distance type and aggregation method selected.

Although Euclidean and Manhattan distances are strongly correlated with network distances, local variations are nonetheless observed, notably in suburban areas. The choice of the aggregation method is also important: accessibility measures computed from census tract centroids, though not inaccurate, yield important measurement errors for 5% to 10% of census tracts. This is especially so in census tracts with lower population density and in those where the land use is largely non-residential. Because the accessibility of health services may be more problematic in suburban areas than in more central urban areas, geographical accessibility studies should be based on the most accurate measures. Thus, using the smallest area unit possible included in the spatial unit of interest appears to be a relevant alternative for minimizing aggregation errors.

Results obtained for Montreal – comparison of the four types of distances and evaluation of aggregation errors – may be generalisable to other North American cities where urban forms are similar. For example, in a study aiming at comparing types of distances, Apparicio and colleagues observed similar results for the eight largest Canadian metropolitan areas [28]. Moreover, results can also be extended to other services and amenities (not only for health services) although the magnitude of aggregation errors may likely vary [2].

Computing accessibility measures in GIS using network distances and more precise aggregation methods is no longer a daunting task. Nowadays, software and modules dedicated to network analysis are effective and user-friendly, notably the Network Analyst Extension of ArcGIS or the TransCad software. Moreover, street network data are easily accessible (for example, Statistics Canada or DMTI data). Because the calculation speed of computers is exponential, the time required for the computation of numerous network distances is no longer a limitation. Consequently, although errors associated with the choice of distance types are important for about 10% of census tracts, we should not avoid using the best estimation method possible for evaluating geographical accessibility. This is especially so if accessibility measures to health services or health-related resources are to be included as a dimension of the built environment in studies investigating residential area effects on health outcomes. Imprecision of accessibility measures could lead to errors or lack of precision in the estimation of area effects on health.

Future studies should investigate the extent of aggregation errors occurring when measuring accessibility to other types of services and amenities, but also in other cities where urban form may differ from that of North American cities, and finally in rural context where geographical accessibility is an important issue.

## Competing interests

The author(s) declare that they have no competing interests.

## Authors' contributions

PA and MA are the principal investigators of the study. They carried out the GIS, statistical and mapping analyses. MR reviewed the literature and wrote parts of the paper. All authors jointly drafted and critically revised the paper, and read and approved the final manuscript.
